# Transactions of the Associated Apothecaries and Surgeon-Apothecaries

**Published:** 1823-12-01

**Authors:** 


*Transactions of the Associated Apothecaries and Surgeon Apothecaries.* Vol. I. 8vo. pp. 640. Burgess and Hill, London, 1823.

[First Analytical Article.]

We are right glad to see this first volume of a periodical repository springing from a base that can hardly fail to furnish ample materials for an annual contribution to the general stock of medical knowledge. The education, professional and scholastic, of the general practitioners is now secured at a standard far above what it ever was before, and perhaps high enough for all practical purposes, and for the great mass of that most useful and hardworking class of medical society. We apprehend that in civilized life, such as it is now constituted, there must always be, in cities and populous towns, a division of labour in the healing as well as in other arts. When the life of man is in jeopardy, the patient and the friends of the patient will grasp at every ray of hope, and will therefore have all the advice which the place or their circumstances can afford. Hence the physician and the operating surgeon must come in for a share of practice in dangerous, and often in trifling cases, be the talents, the experience, or the education of the general practitioner what they may. The elementary education of this last class of medical men, now so much better than formerly, (when the dispensing of medicines was almost their sole employment,) joined to the wide field which they have for observation, must bring all the more talented portion of them on a very near par indeed with the physician. It is in this mixed practice, in fact, where diseases are seen from their earliest stages, and where the effects of remedies are hourly witnessed, that the most solid and useful knowledge can be acquired. Were these advantages unalloyed, the general practitioner would distance the physician on all points of medical science, and the latter class must soon disappear entirely. But the advantages abovementioned are not without their accompanying drawbacks. The harrassing life of the surgeon-apothecary—his broken rest—the multiplicity of his pursuits, (physic, surgery, midwifery, pharmacy, accounts, &c. &c.) and the absolute want of *time*, render it morally impossible that he can reflect on, compare, arrange his observations, so as to reap from them all the advantages which they are capable of yielding. The human mind, in short, is so limited—the range of science so extensive—life sô short, and its cares, anxieties, and necessary avocations so numerous, that none but the highly gifted few can overcome these difficulties, and shine at once in the three great departments of the healing art.

But the surgeon-apothecaries, from the circumstances and for the reasons now stated, are peculiarly well qualified to supply the most valuable and important data on which to ground principles that may ultimately erect medicine into a science approaching to the exact. And if they will but confine themselves to the recording of facts, and to plain observations on those facts, without prematurely framing and wire-weaving hypotheses, they will confer incalculable benefit on science, and ensure the thanks and the veneration of posterity. A work like the present only wants a beginning and publicity to ensure it success—not only in the supply of materials, but in the circulation of them afterwards. It is a duty incumbent on the class of practitioners in question, however, to give timely and efficacious support to the undertaking by contributing communications and encouraging the sale of the volumes as they appear. If they prove lukewarm on *either* of these points, the enterprize is lost. Even if ample materials poured in, the *common sale* of the work will hardly defray the expenses of publication, without the vigorous patronage of the general practitioners. That such patronage will come into action on the appearance of this work we have reason to infer from the burst of encouragement which attended the debût of the London Medical Repository a few years ago. That Journal, contrary to the usual progress and order of things, rose all at once to a high—in fact, to its ultimatum of circulation, an occurrence unprecedented in the annals of periodical literature. True it is, that there existed a fervor of feeling among the general practitioners of 1813, which a lapse of ten years has almost entirely cooled. But we think that to give encouragement to the present undertaking there is no necessity for fervent party spirit, but merely a practical application of laudable zeal to uphold the character and call forth the talents of the profession in general, and of the surgeon-apothecaries in particular.

And now, having done our duty in calling the attention of the class in question to what is essentially their own interest, we shall proceed to make the public at large acquainted with the nature and contents of this primogeniture, which we hope will be followed, at regular periods of gestation, by a long line of healthy and vigorous offspring from the same parent stock.

ARTICLE 1. *An Essay on the Education and Duties of the general Practitioner in Medicine and Surgery; containing Suggestions relating to the Investigation of Disease, and the Registration of Practical Results.* By THOMAS ALCOCK, Member of the Royal College of Surgeons in London.

There are perhaps few practitioners in medicine, whether physicians, surgeons, or surgeon-apothecaries, who have more diligently and successfully cultivated the various branches of medical and other sciences than Mr. Thomas Alcock. This gentleman is one of the favoured few, whose minds are capable of embracing a range of knowledge far beyond what the general run of minds can grasp. In laying down rules therefore for medical education, he has probably calculated by a standard of talent, zeal, and industry, which will be found far too high for general example or imitation. This has been the case with all who have written on the subject of medical education. We appeal to the lucubrations of the late Dr. Beddoes for an illustration. Since the publication of that gentleman's sentiments we dare venture to say that not a single practitioner has gone through the discipline which he represented as necessary for practising the healing art. But it may be said that, by placing the standard high, we give scope to men of all capacities for imitation. Let us suppose that a very tempting apple was suspended from a branch, at such a height that a very expert rope-dancer might, by the utmost exertion of his agility, hope to seize it: is it very likely that the clumsy clowns of the neighbourhood would try more than one or two springs at the object so far above their reach? The application of this simile to the subject in question is sufficiently obvious. General rules should be adapted to the general capacity of mankind. Exalted talents and powerful genius will neither heed nor need them. We shall now, however, endeavour to concentrate some of Mr. Alcock's observations and suggestions on the important subject in question.

We agree with our author that the investigation of diseases requires previous culture of the mind—and also that the acquired powers of the mental faculties resulting from judicious culture "are of more value in the every day concerns of life than the greatest distinctions which Nature usually confers on individuals of the same species."

We apprehend that there is also much truth in the following observation, though it is contrary to popular opinion in general.

"It is not an uncommon error for parents to remain indeterminate regarding the choice of a profession or mode of life for their children: the choice is frequently left to the children themselves, who cannot be supposed to possess the necessary experience to enable them to determine what is really best; they, therefore, often select a profession from some showy and unimportant appendage to it, and never think of the evils inseparable from their choice, until it is too late to benefit by the knowledge. Were parents to determine this point for their children early in life, the mind might, by due care and cultivation, be adapted to the pursuit, so as to ensure a higher degree of excellence than is usually attained." P. 3.

It would be desirable, as Mr. Alcock remarks, that the studies and even the amusements of the youth, designed for the medical profession, should be calculated to improve his powers of observation, of reasoning, and of patient persevering exertion.

"In addition to the ordinary routine of school education, which should comprise grammar, English and Latin, (and Greek, if time permit,) arithmetic, geometry, drawing, &c. every subject of instruction should be perfectly understood by the pupil as far as he has advanced. The writing of a good current hand, accurately and expeditiously, cannot be too sedulously attended to, as indistinctness of character in writing, either in prescribing or in writing directions, may lead to dangerous or even fatal error. The cultivation of a small garden is well calculated to promote industry, patience, and observation; for the preparing of the ground is a necessary preliminary to sowing the seed; and the seed, when sown, does not appear to germinate till after a period more or less remote; whilst the daily attention which various plants require, brings to notice many of the phenomena of vegetation which might otherwise escape notice. The arranging of mineralogical specimens may also serve to promote accuracy of observation. An intercourse with the more simple and useful arts and manufactures will also greatly assist in improving the powers of observation of the young pupil. Occasional exercises in mechanics, in making experiments or models, and in describing accurately the natural or artificial productions which he has observed, will prove useful. But it must be obvious, that however desirable it may be to promote intellectual improvement, yet to form the character of the individual to virtuous habits, to self-control, to unwearied beneficence to others, is an object of still higher importance. The character of Alexander must give way to that of a Howard, who may be pointed to as an example worthy of imitation." 8.

Arrived at the age when school discipline usually terminates, the selection of a master for the youth, is a matter of no small moment. He ought to possess good common sense, a fair share of general and scientific knowledge, and an ardent zeal for the improvement of his profession by all honorable means. "He should not be too young; for early age and inexperience go hand-in-hand: yet, in advanced years, although there may be a large share of experience, that zeal is past which induces the preceptor to share the studies of his pupils, and which is perhaps the most certain mode of ensuring their improvement." Great diversity of opinion has obtained respecting the value of a medical apprenticeship—some denouncing it as time lost; while others consider it as indispensibly necessary, and very profitable. There may be foundation for both opinions. With some masters the time is undoubtedly lost, or nearly so, unless the pupil be possessed of more than ordinary prudence and disposition to study—but with others, the period of apprenticeship (were it not to extend beyond three years) might be profitably spent. Mr. Alcock makes some pertinent remarks on scholastic education; but they appear to be misplaced in a work of this kind:—they should be addressed to those who can rectify the evils complained of, through the medium of some public print of a miscellaneous nature. Here Mr. Alcock enters into minute directions for the conduct of the young apprentice, in weighing out and compounding his medicines, writing clear directions on the labels, studying the materia medica, arranging his pocket instruments, dressings, apparatus for *post mortem* examination, &c. all which may be perused with great advantage by the tyro. The following passage we recommend to the attention of the master.

"It has been a general rule to require from my domestic pupils a written report of every dissection at which they were present, as a sine quâ non of their attendance, and that the report should be delivered to me within the twenty-four hours next ensuing. At first, as might be expected, these reports were very imperfect; but, as by repetition the subject became better understood, they gradually improved. The imperfections and occasional errors were pointed out, and sometimes the statement which I had made for my own use was referred to, to show the omissions in those of the pupils, and further that they should be aware that it was their welfare, and not my convenience, which made me exact the strict performance of this exercise." 21.

The plan Mr. Alcock has found to succeed best was to avoid teaching too much at once, and to endeavour to make the little taught well understood. Thus, in opening bodies, "the alimentary canal may form a first lesson, naming the parts simply without any reference *to technical jargon.*" We really do not understand how the parts of the human body can be better taught in simple than in technical terms. Would Mr. Alcock begin thus—the gullet, (not œsophagus)—the paunch, (not stomach or ventriculus)—the twelve inch gut, (not duodenum)—the empty gut, (not jejunum)—the gut like an eel, or twisted gut, (not ileum)—the dung gut, (not colon) the straight gut—which, by the way, is a very crooked gut, (not rectum.) Thus, instead of simplicity, we would have vulgarity—and instead of classical and concise terms, we should have inelegant and butcher-like phrases. We make this observation, because Mr. A. seems to sneer, in many places, at scholastic terminology and arbitrary distinctions, without which the study of medicine would be difficult, and its literature barbarous. Mr. Alcock goes on through many pages, describing the minutiæ which the master is to be constantly overlooking in the progressive steps which the pupil is making in all the different branches of the profession. But we appeal to every surgeon-apothecary in this kingdom, who has a common run of practice, and a wife and family to attend to, whether one-tenth of the discipline which Mr. Alcock proposes can be superintended by the master? Can such domestic homilies as the following be reasonably expected after a man comes home jaded, fatigued, and care-worn with the daily duties of his profession?

"I have known the master join in a family circle, in evening amusements calculated to improve his pupils, on a footing of perfect equality. Each took a subject of elementary knowledge which he had previously studied, and communicated it to the others, in the form of a short and familiar lecture. The communication was guarded from interruption; but as soon as it was finished every one in turn was permitted to ask questions relating to the matter, or to supply any illustration that might have been omitted. One of the juniors acted as secretary, and registered the results of the discussion. The exercise was both delightful and profitable." 28.

Now this is all very well for Mr. Alcock, whose *family circle* consists entirely of his pupils. But if he had a wife and half a dozen children, he would find the business different. Mr. Alcock should have consideration for his brethren, who have neither the time, the talents, nor the means of making themselves not only the masters, but also the private tutors of their apprentices. Many young men will read the system of education proposed and acted on by our author—and most of them must draw comparisons by no means favourable to their masters. We do not say that Mr. Alcock's plan of medical education, during apprenticeship, is Utopian, as far as he himself is concerned; but we appeal to the general sense of the profession, whether it is not Utopian for nineteen-twentieths of his brethren. This, in fact, was represented to Mr. Alcock before the MS. went to press. "A friend, to whom the preceding observations were shewn, objected, that such a plan of instruction would require the sacrifice of nearly the whole time of the teacher." And truly so it would. It is in vain that Mr. Alcock assures us, "from experience," that this is far from being the case. He draws from the experience or example of an individual, and consequently his precepts are inapplicable to the great mass of general practitioners.*

Mr. Alcock thinks, that the advantages of attending lectures, during apprenticeship, have been much over-rated. He has always, however, permitted his pupils to attend anatomical lectures and dissections previous to the expiration of their apprenticeship. Surely it would have been equally as desirable that *chemical* lectures should have been attended to in that period, since they store the young man's mind with knowledge which is every hour put in requisition in his daily employments. We agree with our author, that the plan of allowing the youthful pupil to commence the whole routine of lectures and apprenticeship at the same time, will generally fail—especially if he be designed for a surgeon-apothecary. In the case of some of his friends, under this plan—

"The pupils have wasted their time, and, after repeated courses, have remained ignorant of the most simple principles of medical science: whilst habits, the very reverse of diligence, were established." 33.

The apprenticeship over, the elementary education of hospitals and public lectures becomes an important subject of consideration. The following picture deserves attention, and contains unpleasant truths.

"The prevailing error, with many students, seems to be, to consider the passing of examinations as the great object to be aimed at, rather than the acquirement of that knowledge without which they cannot perform the duties of an arduous profession with honour to themselves and with benefit to their patients.

"A student arrives from the country, after having served an apprenticeship, with opportunities of gaining professional knowledge, more or less limited, according to the circumstances in which he has been placed. He arrives in the metropolis with a determination to remain one year, and to pass his examinations at Apothecaries' Hall and at the College of Surgeons. He makes inquiry, and finds that the examination at Apothecaries' Hall requires certificates of attendance on two courses on anatomy and physiology; two courses on the theory and practice of medicine; one course on chemistry; one

---

* That Mr. Alcock has practised every thing he preaches we have the very best reason to know; and such is the high opinion which we entertain of this gentleman's talents, acquirements, and integrity, that had we a son to put out as an apprentice to-morrow, there is not a man within the whole circle of our acquaintance, (which is not very limited,) whom we would put in competition with Mr. Alcock as the master, guardian, and preceptor of that son. After this sincere declaration, Mr. Alcock cannot surely apply our strictures to himself personally, but to the too rigid system of instruction which he would impose on those who have not his talents to perform them.—*Rev.*

on materia medica; and six months on the medical practice of an hospital; lately, I believe, restricted to the certificate of some branch of the College of physicians in London—total, six courses and six months' medical hospital attendance. The College of Surgeons requires now three courses on anatomy, and twelve months' attendance on the surgical practice of a metropolitan hospital. *No country hospital!* Lectures on surgery are required: but the number of courses is not stated, say two. The total for the year's employment will stand thus:—

| | |
|---|---|
| For Apothecaries' Hall . . . . . | 6 courses. |
| For College of Surgeons . 5—2= | 3 |
| Total | 9 courses; |

and six months' medical and twelve months' surgical practice, without any allowance being made for practical dissection, or for midwifery.

Leaving Sunday out of the calculation, let us examine the time these courses of instruction occupy.

| | |
|---|---|
| Anatomy.—Lecture from one to two hours, according to the custom of the school } say | $1\frac{1}{2}$ |
| Demonstration . . . . . . . . . . . . . . . | 1 |
| Practice of Physic . . . . . . . . . . . . . | 1 |
| Chemistry . . . . . . . . . . . . . . . . | 1 |
| Materia medica . . . . . . . . . . . . . | 1 |
| Medical practice . . . . . . . . . . . . . | 1 |
| Surgical ditto . . . . . . . . . . . . . . | 1 |
| Lectures on surgery twice or thrice a week . . . | $\frac{1}{2}$ |
| Total | 8 |

This calculation does not include the necessary time lost in going from place to place, which is very considerable, nor that which must be taken for meals: so that from eight in the morning till dusk in the winter months, does not, without some of the above being omitted, afford one single hour for practical dissection, for midwifery, botany, or any other accessory branch of science; or for what is essential to form a sound judgment, viz. for reflection on what has been seen and heard. Some teachers of physic, knowing the importance attached by students to passing examination at the Hall, kindly condescend to *cram* their pupils for an additional hour daily, under the name of examination. So much does the taste for this parrot-like exercise prevail, that the majority of pupils would rather absent themselves from any or all of their other pursuits, than be absent from this delectable treat. During the short interval allotted to what are called the summer courses, the time may not be so fully occupied; but the heat of summer does not constitute the most favourable time for dissection; nay, at the schools of anatomy connected with the larger hospitals, no lectures on anatomy are delivered during summer, neither are the dissecting rooms open. To hurry, thus, through a course of studies, if studies they may be called, as too frequently happens, is not less absurd than the attempt to fill a sieve with water, in which the more that is poured, so much greater is the rapidity with which it escapes." 36.

With so imperfect a foundation it is indisputable that many young men return to the country with little more than the merely nominal qualifications of a surgeon and apothecary; but it is to be hoped that this confined and deplorable plan is fast falling into disrepute. From the foregoing observations it may be gathered that Mr. Alcock does not consider one year sufficient for the London studies of a young man let loose from his apprenticeship—unless that apprenticeship has been conducted in the manner recommended by Mr. Alcock, which we may safely venture to affirm will never take place out of Mr. Alcock's own house. But even under the most advantageous circumstances our author does not recommend so limited a period as one year. "The arrangement should be made for *at least* two years, whether these years be in immediate succession, or separated by an interval, during which some share of the responsibility of practice may be entered upon, or the mind expanded by the acquisition of general knowledge."

Mr. Alcock justly observes that anatomy is the ground work of all. The student, therefore, should not only begin early to dissect for himself, but he should become master of osteology before he comes up to town at all. This, any youth, with the bones and Monro's description of them, may easily do. Mr. Alcock recommends the student to get early into the habit of sketching the bones—a practice which compels to a more accurate inspection of them than would otherwise take place.

Osteology completed, the student should take an extremity, and dissect, at first, with reference to the bones and muscles only. Every muscle dissected, should be compared with its description, in some good work, and every attachment should be traced with care. Plates will assist him when at home—but they are very imperfect auxiliaries. For other minute directions in the study of osteology and myology, we must refer to Mr. Alcock's paper. The knowledge of the internal viscera is now to be attained, or rather improved; "for if the apprenticeship have been fairly conducted, these viscera must have been well understood in *all essential points*, although the *minutiæ* may have been passed over." Does Mr. A. mean by this that a knowledge of the *minute structure* of the viscera is not among the *essential points* of information in anatomy? without knowing, at a glance, the minute healthy structure, pathology can never afterwards be studied with advantage; and we ask if, under ordinary circumstances, cheap prentice of an apothecary can be expected to acquire a knowledge of the minute structure of the internal organs?

Mr. Alcock gives many judicious and admirable instructions on the mode of conducting the anatomical studies, which deserve the student's best attention, summing up the arrangement as follows:—

"The general arrangement for the first season will be confined to—

Anatomy and physiology, the chief object.
Natural philosophy, as recreation.

The dissections will stand thus:

Half of a subject for the bones, muscles, and joints.
Ditto for the injected arteries.
One small subject for nerves.
One entire head and neck for injected veins, &c.
Half subject for full dissections, without injection.

"The internal viscera may be examined in each subject.*

"The time may be stated as *at least* four hours per day, six days in the week.

| | |
|---|---|
| "For the study of the bones, before the commencement of dissection . . . . . . . . . . . . . | four weeks. |
| For the muscles, ligaments, and joints, allowing a fortnight for the head and neck, and the same for each extremity . . . . . . . . . . . . . | six weeks. |
| For the injected arteries . . . . . . . . . . . | six weeks. |
| For the small subject for nerves . . . . . . . | two weeks. |
| For the injected head and neck . . . . . . . | two weeks. |
| For the full dissections, without injection, allowing three weeks to each division, as above . . . . | nine weeks. |

"Total, twenty-nine weeks, leaving three weeks for the dissection of the internal viscera, the organs of sense, &c.

"The day work will stand,

| | |
|---|---|
| For practical dissection, and making studies from dissected parts . . . . . . . . . . . . . . . . | 4 hours. |
| For attendance on anatomical demonstration . | 1 hour. |
| For anatomical lecture . . . . . . . . . . . . | 1½ hour. |
| For reading, arranging tables, &c. . . . . . . . . | 2½ hours. |
| | 9 |

"Total, nine hours, exclusive of lecture on natural philosophy, one hour twice a week." 48.

On this arrangement we would take leave to remark that, excepting under very peculiar circumstances where the student had three or four years to dedicate to elementary mat-

"* The expense will be somewhat less than that of two entire subjects."

ters, nine hours a day, for the whole of the first season of 32 weeks, is too great a proportion of time for anatomy alone. Nor do we think this plan very consistent with the preservation of health, in a young man just arrived from the country, and spending six hours and a half daily among the putrid exhalations of a dissecting room and anatomical theatre. Few ever prosecuted their anatomical studies with more enthusiasm than the writer of this analysis; but his health was materially injured by an application of four hours a day to dissection, though his constitution, at that time, was vigour itself. We believe, that if three or four hours daily are dedicated to anatomy, and that continued through every session of attendance in London, there will be more knowledge acquired at a smaller sacrifice of health, than on the plan which Mr. Alcock has laid down.

Mr. Alcock advises, (and we think the advice judicious,) where time will permit, that the student should go into the country during the summer, rather than prosecute his studies through the dog days of London. In the country he may study botany, (but Mr. A. has not yet allowed him to attend any botanical lectures,) and recruit his anatomical and physiological memory by the aid of books and plates. In recommending the medical student to make himself familiar with mechanical operations, and edged tools generally, our author takes occasion to deplore the aukwardness and clumsiness of surgical operations "compared with manipulations in other arts." He instances a surgeon, who set about sawing off a leg, the saw being defective, "owing to the teeth not being *set*;" in consequence of which, the saw became jammed in the bone, and, like a restive mule, would neither move backwards nor forwards. If we remember right, the principle of surgical saws is, that the blade shall gradually become thinner from the teeth towards the back, which simple principle completely obviates the danger of the instrument getting jammed as it proceeds to any depth in the bone, even if the teeth are not set off obliquely from the plane of the blade. Both precautions, however, are best.

The summer over, and the pupil returned, a severe course of clinical study is to be commenced. Mr. A. dissuades from taking notes during the first season. To us the propriety of taking notes at any season is very doubtful. It was our own practice to go directly home and write down an epitome of the lecture from memory. By this plan three useful objects were attained. In the first place we lost no portion of the lecture from the distraction produced by taking down notes. In the second place, it induced a habit of accurate *attention*, the very basis of memory. In the third place, it induced a turn for concise and terse composition, which has been of no small advantage to us in after life. Having given minute directions how to take notes after the first course, Mr. Alcock concludes with the following observation, which certainly does not hold out much encouragement to the practice which he had just been recommending.

"But he who lays a solid foundation in elementary studies, will find that though his shelves may be filled with his volumes of manuscript lectures, which bear honourable testimony of his diligence; *yet years of practice roll away without his ever finding it necessary to refer to them;* whilst the reminiscences drawn from his own actual observation of disease, give him a well-grounded confidence, which mere lectures and the lessons of others can never impart." 55.

Chemistry is now to be entered on, and much deserved eulogy is heaped on that delightful science. We think, however, that throughout this paper there is too much prosing illustration, of a kind that might be very appropriate for domestic and familiar instruction to pupils, but which becomes rather tedious in a didactic composition addressed to the profession at large.

After eulogising the excellent lectures of Mr. Brande,(which indeed are above all praise) the expertness of Mr. Farrady, and the admirable apparatus of the Royal Institution, Mr. Alcock goes on to the praises of another distinguished ornament of the profession, who has ever stood high in our estimation. In our humble opinion, however, friendship should be temperate in its eulogies, lest it injure the object designed to be benefited. Those who are best acquainted with the actual state of medical knowledge, will hardly believe that even the genius of an Armstrong (and we admire his genius) has yet reduced medicine to "a *demonstrative science.*"

"The theory and practice of physic have been too long disgraced by *technical jargon and artificial distinctions* which do not exist in nature, and which, to say the least of them, are useless at the bedside of the patient. To attain correct elementary ideas of the nature of disease, is of the highest importance, as in a great degree influencing the future usefulness of the medical practitioner; and without disparagement to other lecturers, I may be permitted to observe, that the zeal and ability which have characterized the labours and raised the reputation of Dr. Armstrong, have never been more successfully exerted than in his lectures.

"These lectures go far to reduce the theory and practice of medicine to the simplicity of a *demonstrative science*, and to substitute accurate observation in the place of vague hypothesis; and if the minds of the pupils be duly prepared by adequate elementary education, to profit by these excellent lessons, which contain the results of

Dr. Armstrong's unpublished practice, the benefits they are designed to diffuse cannot fail to be realized." 58.

Knowing, as we do, the talents and observation of Dr. Armstrong, we cannot but strongly recommend his lectures to the attention of the student, and hope that no private emolument will prevent his soon laying before the public at large the substance of those lectures; for we believe that medicine is any thing but a demonstrative science in the hands of others.

Mr. Alcock inclines to a reprobation of the cruelty exercised in experimenting on animals. We honour his feelings, but we cannot quite participate in them. We have no idea of a sentimental lachrymation over a decapitated rabbit, while the poor turtle is kept three months *dying* on his back (without the least commiseration) under a tropical sun to glut the paunch of a luxurious alderman.

Speaking of the neatness and dexterity which a student should endeavour to acquire in bleeding and other subordinate operations, Mr. Alcock adds—

"It is not twenty-four hours since a medical friend adduced an instance which occurred a few days ago. He had witnessed the dissection of a patient, who had gone to an hospital to be blooded, and who had died in consequence of that simple operation. *This fatal termination, he observed, could only result from extremely bad management;* the unfortunate patient left a widow, without the means of supporting herself. A student, who was present at the above relation, stated, that at the hospital which he was attending, it was a general remark, that scarcely one of the out-patients who were bled, escaped without the arm festering." 60.

We cannot commend the spirit or the judgment of the friend who passed the above severe sentence on a public hospital. Most men of observation know, that in certain seasons, and in certain individuals or habits of body, every scratch festers, and every lancet-puncture inflames, even if made by a Cooper or a Cline. We remember being placed in a situation, about ten years ago, amid the unhealthy marshes of Zealand, where pneumonia prevailed to a great extent, and where almost every man who was bled had an inflamed arm, and sometimes of a very formidable character. At first we thought it was owing to negligence or bad lancets of the assistants, and then bled with our own; but with no better success. We soon indeed found, that such was the nature of the atmosphere at the time, that almost every scratch inflamed and brought on an erysipelatous affection. Would it not have been equally cruel and unjust to blame these occurrences on the medical practitioner?

The time which Mr. A. recommends for hospital attendance, during this second season, is two hours daily, comprising both the medical and surgical practice. He cautions the pupil, (but if Moses were to return upon earth and preach to them on this subject he would not be listened to) against running after extraordinary cases and operations, in preference to the every day duties of the surgeon. But how can we wonder at the pupil's curiosity, when we see grey hairs running to an hospital once in a year to see some extraordinary operation, although their trembling hands, and still more trembling hearts, would not permit them to take a scalpel in their fingers on the most trifling occasion.

"The pupils' fees required and received for hospital attendance in London are sufficiently large to warrant the expectation that the physicians and surgeons should devote a very considerable share of attention to the instruction of their pupils. Whether these expectations be generally realized, or disappointed, I must leave for others to determine: 'Palmam qui meruit ferat.'" 61.

Many of the medical officers of our hospitals certainly do pay considerable attention to their pupils, but the want of clinical lectures renders the hospital attendance of the great body of students a mere dead letter. A short train of clinical observations on the principal cases would attract the attention as well as the attendance of the pupils, whereas the routine is now so dull, that perhaps not a third of the young gentlemen are regular in their attendance—and still more would absent themselves, (thinking the time better employed in grinding,) were they not afraid of their features becoming remarkable, from want of familiarity, and consequently that they would be liable to notice on the part of the surgeon who was to grant the certificate at the end of the year.

Mr. Abernethy's surgical lectures are highly lauded by Mr. Alcock—"but not with any view to disparage the lectures of any other surgical lecturer." We deem it a very delicate point, however, in a public writer, to single out an individual lecturer, and laud him to the skies; for surely it is a mere farce to turn short round to the others and say—"Gentlemen, because I aver that Dr. or Mr. so and so is the very *best* lecturer in this metropolis—I do not, of course, mean to say, that he is a *better* lecturer than any of you."

We shall pass over Mr. Alcock's keen satire on the common practice of his brother practitioners in suspending their certificates for public view in their shops or dispensaries. Considering the number of irregular practitioners which infest town and country, there is some excuse for the surgeon-apothecary, who has gone through his regular studies, in thus proving, by authentic documents, that he is legally and properly qualified to practise his art. After he is completely established and known, he, of course, has no occasion to exhibit these testimonials of professional education. We think Mr. Alcock's episode of Mr. Abernethy's pupil, in pages 63, 64, and 65, would be just as well omitted in the next edition of these Transactions.*

"It may not be amiss to recapitulate the employment already proposed for this second season, Chemistry, one hour thrice a week—Botany and materia medica, ditto—Theory and practice of physic, ditto—Practical and pathological dissection, one hour daily—Anatomical lectures, one hour and a half daily—Medical hospital practice, one hour daily—Surgical hospital practice, one hour daily—Surgical lecture, one hour, twice or thrice a week.

Total, four lectures thrice a week=2 hours daily.
*Anatomy*—Dissection, 1 hour—Lecture, 1½ hour=2½ hours.
*Hospital*—Med. practice, 1 hour—Surg. prac. 1 hour=2 hours.

6½
Remaining for reading, writing, meditation, &c. . 2½

Total of intellectual labour 9 hours." 65.

Mr. Alcock thinks that the lectures at the College of Surgeons occupy less time than ought to be devoted to the illustrations which the splendid museum attached to the College requires.

"The burlesque of Molière's Malade Imaginaire becoming doctor himself, is not more preposterous than the idea that all that is important in surgical education can be compressed into the discourses of twenty-four hours, or even of double that extent." 66.

The liberality of the College of Physicians, in throwing open their lectures to the whole of the profession, is praised. We have not the smallest doubt but that these lectures will be as numerously attended as those in Lincoln's-Inn Fields, when they come to be delivered in Pall Mall East, instead of the murky atmosphere of Warwick Lane, impregnated with the effluvia of incarcerated human beings on one side, and the reeking offals of animals on the other.

The student has now terminated his second season, and his health will probably require the renovation which country air can so well bestow. In this interval of relaxation, while enjoying the pleasures of a rural excursion, the perusal of

* As we think there is a very great deal in Mr. Alcock's paper which would be exceedingly useful for students, we advise him to print it in a small and cheap form for their use, after the Transactions have had a fair run. Perhaps, too, he may revise and modify some of his opinions.

works treating on medical ethics, as those of Percival, Gregory, &c. would be beneficial in confirming the ardour and devotion of the student to the duties of the profession he has chosen. Would to God that medical ethics were more studied and practised than they are! Till a thorough and general conviction of the utility of rigid adherence to the rules of medical ethics obtains, we despair of seeing our profession on a par with the other learned bodies distinguished for their amenity and liberality towards each other.

Our author advises the pupil to visit, during his recess after the second season, the medical schools and hospitals of Dublin, Edinburgh, and Glasgow, not neglecting the various country hospitals and other medical establishments. It is after the third season that he advises a trip to Paris, neither the time nor the expense being greater than that of going over to Dublin. He may go now for 48 shillings to the capital of the Great Nation. Even if he only stays *a week* there it will be a great advantage to him all his life—inasmuch as nothing need be new or great, or difficult, or meritorious, afterwards among his brethren at home—for all these and more he had seen in Paris! But, irony aside, a student of good sense and laudable curiosity, may be nothing the worse for a short visit of three or four months to the Continent. Considering the difficulties under which anatomy labours in this country, the facilities of dissections in Paris are great temptations to pass some time in that capital.

Our author regrets that some of our excellent country hospitals should be deprived of the advantage of granting certificates of qualification for examination at Surgeons' Hall. There can be no doubt that a pupil, brought up at a good country hospital, is infinitely better informed in his profession than the great mass of pupils who run or shun the metropolitan establishments. But it would be invidious to draw a distinction, by conferring privileges on some country hospitals, and it would, perhaps, be dangerous to extend them to *all*. Here it may be proper to promulgate the latest regulation issued by the Royal College of Surgeons respecting the candidate for a diploma.

" '*Royal College of Surgeons in London. Court of Examiners: 1st day of January*, 1823.

" 'Candidates for the diploma will be required to produce, prior to examination, certificates—

" '1. Of having been engaged six years, at least, in the acquisition of professional knowledge:

" '2. Of having regularly attended THREE courses, at least, of anatomical lectures, WHICH HAVE BEEN DELIVERED DURING THE

WINTER SEASON; and, also, one or more courses of chirurgical lectures; in London, Dublin, Edinburgh, or Glasgow:

" '3. Of having performed dissections during two or more courses, IN THE WINTER SEASON, in London, Dublin, Edinburgh, or Glasgow:

" '4. Of having regularly attended, during the term of, at least, one year, the chirurgical practice of one of the following hospitals, viz. St. Bartholomew's, St. Thomas', the Westminster, Guy's, St. George's, the London, or the Middlesex, in London;—or the Richmond, or Steevens' in Dublin;—or the Royal Infirmary in Edinburgh;—or the Royal Infirmary in Glasgow:

" '5, And of being twenty-two years of age.

" 'Candidates under the following circumstances, and of the required age, are, also, admissible to examination.

" 'Members of any of the legally constituted Colleges of Surgeons in the United Kingdom.

" 'Graduates in medicine of any of the universities of the United Kingdom; who shall have performed two, or more, courses of dissection, as above specified; and who shall have regularly attended, during the term of, at least, one year, the chirurgical practice of one of the above-mentioned hospitals.

" 'The above rules are required to be observed by candidates to be examined for the testimonial of qualification of principal surgeon in any service.

" By Order, EDMUND BELFOUR, *Secretary.*

" 'Candidates are to observe that tickets of admission only, will not be received as certificates or evidence of attendance.

" 'The late regulations, denoted by small capitals, are not designed to operate retrospectively; therefore, persons who have attended two or more courses of anatomical lectures prior to the 1st of January 1822; or who have attended three or more courses of such lectures, although they may have been delivered during the summer months, prior to the 1st of February, 1823; or who have been engaged in the study of the profession five years prior to the date of these regulations, will be admitted to examination as heretofore.' " 73.

Mr. Alcock considers it a great hardship on some students and some teachers that the courses of lectures on anatomy should be restricted to the *winter season*. But Mr. Alcock must know that there are *reasons for all things*. The following observations are not a little pertinent, and we imagine the queries could not be satisfactorily answered—excepting upon *corporation principles*, which have the wonderful power of reconciling all anomalies, and obviating all objections.

" It may, however, be assumed, that the *verbal* examination at the College of Surgeons either *is*, or *is not*, a sufficient test of surgical ability. If the former be admitted, that such examination *is* a sufficient test, what can be the necessity for vexatious restrictions? Is it not sufficient to determine whether the candidate actually possess the knowledge and ability necessary to perform the duties of surgery?

"If the candidate really possess the necessary knowledge and ability, what, in the name of common sense, can it signify, whether his knowledge has been acquired in summer or in winter, in provincial or in metropolitan hospitals? Will his knowledge be less efficient in the cause of humanity, on account of its having been drawn from the one source or from the other?

"But should any one be driven to the conclusion that such examinations *are not* adequate tests of practical knowledge and ability, let him uphold that the same number of hours applied to dissection in a clear summer's morning are far inferior to the like number in a dull foggy day in winter; and that there are no sources of surgical knowledge, save and except those few particularized by the Court of Examiners!" 74.

The student is now returned to the metropolis for the third season or year, with renewed health, "and a sufficient enthusiasm to overcome every surmountable obstacle." On a rigid self examination, before he enters on the third course of studies, the student will probably find that his physiological knowledge is imperfect—that notwithstanding he has diligently attended the instructions of his medical and surgical teachers, yet that at the bed-side of the patient he is, not unfrequently, utterly at a loss to make his nosological definitions and the symptoms agree—some being absent and others superadded—so that he is bewildered.

"With all his instruction, he has never been taught to investigate disease so as to represent clearly to his own mind, what are the parts or organs of the body which actually deviate from the state of health; what is the nature of the derangement which they suffer; nor to deduce, from these simple facts, principles of treatment rationally adapted to the restoration of health! He has indeed witnessed many cases of recovery to take place without any rigid investigation of the nature of the disease, for the salutary efforts of nature are, in many instances, sufficient to effect recovery without assistance from art; but it is also probable that he may have witnessed, with sorrow, in examinations made after the death of the patient, that the disease had not been, during the *life* of the patient, understood; that the disease was in its nature remediable; and that probably a greater degree of diligence in investigation, aided by the resources of general and medical science, might have led to the discovery of the nature and extent of the malady, in time to preserve the life of a fellow-creature." 75.

Mr. Alcock justly observes, that were *post mortem* examinations more universally instituted, much improvement in the healing art would be the result. "But many there are who would rather that the grave should conceal the errors of their practice, than that those errors should be exposed, either for the improvement of themselves or others." From a pretty considerable intercourse with general practitioners we can confidently assert that there are comparatively few (of the juniors at least) who are not as anxious for post mortem examinations as Mr. Alcock could desire them to be. Some of the elder branches, indeed, or of the old school, are very lukewarm in the cause of morbid anatomy—and when they do bring themselves to examine a head, chest, or abdomen, make quick and sad work of it! But these things are on the wane; and not only a far better education, but a far greater spirit of investigation pervades the general mass of medical society now than formerly.

Our author gives very minute and very excellent instructions to the student and young practitioner, respecting the mode of investigating diseases at the bed-side of sickness—and this section we particularly recommend to them, as it is of the very utmost importance to have some regular plan of proceeding, otherwise much time is lost—many repetitions are made—and, after all, but an imperfect case obtained. We have often, indeed, been astonished at the blundering, confused, and irregular manner in which physicians, even of reputation, have conducted the investigation of an obscure case. The mode recommended by Mr. Alcock is too tedious and complicated for either general practitioners or practitioners in general; but it is a very good model for the student, and for him who is only commencing practice. Our own mode is to examine severally the functions of the great organs—for instance, the function of respiration, of circulation, of secretion and excretion, digestion, &c. and then, after getting a short history of the case, to examine, if necessary, how far the great viscera of the abdomen or thorax be organically affected, by means of abdominal pressure and thoracic percussion. By a systematic mode of proceeding, even a complicated case may be sufficiently investigated, in general, in twenty or twenty-five minutes.

Mr. Alcock considers the stethoscope of Laennec as a valuable instrument in diagnosis; but properly observes that, like all other instruments, it requires a little time and practice to become acquainted with its merits.

"In several instances in which the symptoms were such as to leave no doubt in my mind that enlargement of some of the great blood-vessels existed within the thorax, I have been enabled to trace the extent of derangement more accurately by means of this instrument; and it has been a source of confidence both to myself and the patient to be able to ascertain from time to time that diminution of preternatural pulsation (under the use of such remedial means as tended to diminish the force of the circulation,) which marked the progress towards returning health. It is a dangerous error to consider such cases as hopeless; if they be carefully investigated, it will generally be found that they originated under circumstances, which excited the heart's action to an extent more than equal to the powers of resistance in the artery. Let timely attention abate the cause, and the effect will gradually subside. Even in cases in which the enlargement was too obvious to be doubted, and the humble stations in life of the individuals have precluded that absolute rest which forms one of the best remedial means; yet by watching and early treatment under any increase of symptoms, years have passed away in the enjoyment of a very tolerable state of comfort." 90.

The danger of trusting to a single symptom, or even to two or three leading symptoms, is strongly pointed out by our author, and illustrated by examples. Thus he has known a physician mistake aneurism of the aorta for laryngitis, the former disease, by pressure on the trachea producing a train of symptoms resembling the latter. Speaking of the effects of mind on matter, or, more strictly, of mental emotions on the physical organization, Mr. Alcock instances a case of compound fracture in a public hospital which was going on well, till, in an unguarded moment, an ignorant pupil exclaimed, in the patient's hearing, that the limb could not be saved. The sudden effect of the depressing passions was such, that every thing took an unfavourable turn, and the patient died. A table of items of enquiry in the diseases of children is given at page 95, which we recommend to the attentive study of the young practitioner.

Mr. Alcock very properly endeavours to draw the attention of both young and old to the state of the air passages in various diseases. The mucous membrane of the lungs has been still more neglected than the mucous membrane of the intestines, in pathological dissections; though there is more necessity for the investigation, and less excuse for the omission, as it is unattended with the inconvenience inseparable from opening the long line of alimentary canal. The function of respiration being more essentially necessary to life than even digestion, it is evident that interruptions to this important process in diseases must have a greater and more sudden effect than the interruption of any other process, with the exception, perhaps, of the circulation. We agree entirely, therefore, with our intelligent author, that examination of the air passages ought to constitute an essential part of every anatomical investigation of morbid appearances.

"One out of numerous instances may be mentioned. An elderly man laboured under fracture of two of his ribs. A bandage had been applied not only to the chest, but also surrounding the abdomen; and this bandage had been kept wet for some days, by lotions.

Bronchial inflammation, as might be expected under such circumstances, supervened, and the patient died. Inspection after death was permitted, to which a surgeon who had seen the patient once during his illness, was invited. He arrived just as the examination was said to be finished, but without any satisfactory result; there being none of the common marks of inflammation of the chest (pleura,) and the abdominal viscera being tolerably healthy. He inquired what was the state of the air-passages? They had not been looked at. He requested leave to examine them, and found the bronchia and their ramifications filled with fluid of a puriform appearance. The lungs were loaded with similar fluid, which copiously exuded, mixed with a portion of air, on pressing any part of which a section had been made. The bronchial lining was much inflamed, but there was no ulceration. Thus the unexplained cause of death became apparent!"

The third season over, the student should arrange the materials which he has collected or observed. This will be a pleasing and useful exercise, and will enable him to generalize the facts, to compare the results of the various modes of treatment, and thereby to establish that method which has proved the most entitled to confidence. Mr. Alcock properly observes, that the study of books may be of great assistance, for although "experience can never be acquired from reading, it may be prompted and rendered of much easier acquirement by its aid."

"It is not enough that the actual state of the science be already ascertained; but the progressive improvements in medicine and the accessory sciences, which every year brings forth, should be added to the previous stock of information.

"I would caution the student against contenting himself with superficial views and mere dictionary knowledge. Without wandering from one subject to another, whilst he has any disease under observation, let him study it profoundly; let his reading be directed to that particular point; let him examine and compare the descriptions of others with the symptoms which he may observe at the bedside, and let him determine how far the reasonings correspond with the facts adduced." 101.

The pupil may now present himself without fear at the College of Surgeons and Apothecaries' Hall; "but let not the passing of examinations paralyze the future exertions of the student in improving his knowledge of the healing art. However zealously he may labour—however ample his opportunities, yet will there be sufficient scope for improvement. The longest life of the most highly gifted individual would not suffice to supply all the desiderata in medical science, or to make any very near approach to perfection."

Mr. Alcock now glances at "the artificial divisions of the medical profession," but refers us for a proper appreciation of this subject to the well known discussion published a few years ago in the Edinburgh Medical and Surgical Journal. From the following law laid down by the Edinburgh writer, and quoted with approbation by our present author, it appears that physicians and surgeons are mere excrescences from the trees of medical knowledge, and depending on *contingencies* only, (if they mean contingencies of sickness, wounds, &c. they are right,) for maintaining a separate existence."

"From all the foregoing statements and considerations, it must be manifest, that this practitioner *more perfectly represents the medical character than any other;* that, in fact, he alone can be identified with the profession, of which *they who compose the other departments are but partial members, formed into separate associations by casual influences, having no claim of abstract right to that superior, and almost exclusive countenance and protection which they have hitherto engaged, and dependent on contingencies only for maintaining a separate existence.*

" 'With respect to the qualifications of the general practitioners, it is requisite that they be *fully competent to the practice of physic, surgery, midwifery, and pharmacy,*—in fact, to *every thing* that medical science and practice can be supposed to extend to." 104.

We would ask the Edinburgh and the London writers what it is that renders it "*requisite*" that the general practitioner should have a perfect and intimate knowledge of medical science in every department? Is it the existing regulation which prescribes one year's study in London previously to passing Apothecaries' Hall? But set the prescribed regulation aside, let us look, not at what *ought to be*, but what actually *is* the state of the case. Let us go to Apothecaries' Hall, and to the College of Surgeons on examination days, and we shall find at least one half of the candidates, who cannot shew certificates of one hour more than the prescribed period of attendance on hospitals and lectures. And we would seriously ask the writers and approvers of the foregoing extract, whether they could conscientiously assert that such candidates are perfectly qualified to start as physicians or operating surgeons, and that it is merely an artificial distinction, having no foundation in abstract right or justice, which separates them from the very highest orders of the profession? This is bringing the decision of the question to the tribunal of plain demonstrable facts, and not of speculations. If the education of the surgeon-apothecaries of Great Britain can be brought to the state of perfection contemplated either by the Edinburgh writer or by Mr. Alcock, why then we have no hesitation in saying that the office of physician or surgeon is perfectly unnecessary, because no man can be *more* than "fully competent" in every branch of medical science. Hippocrates himself was no more—nay not so much, for he could not perform the operation of lithotomy. But a more Utopian expectation never entered the mind of man, than that which contemplates a perfect equality of education and study among all ranks of the profession. It never can take place till there is an equality of rank, condition, and circumstances throughout society in general. The remuneration accorded by the middling and lower orders of the community to the surgeon-apothecary for his professional services, never can be such as to repay the expence of an education such as the physician is forced to have—and consequently, there must be gradations of rank and qualification in the profession, to meet the gradations of wealth and rank in society at large. We are well aware, indeed, that there are almost innumerable instances of splendid talent, extensive acquirement, and consummate skill, among the general practitioners of this country. But these instances more frequently result from great native capacity in the individuals, conquering all difficulties of time and place, than from expensive and elaborate educations, scholastic or medical.—Were it not invidious to appeal to living examples, we could prove, that many of the most distinguished ornaments of medicine and surgery, at the present moment, had originally an extremely imperfect education, whether we look to literature or science. It will be sufficient to point out one of the illustrious dead—JOHN HUNTER.

In examining the state of the general practitioner, Mr. Alcock takes occasion to deplore and expose the incongruities and defects of the late act—*in their favour*—if that act can be called in their favour, which exposes to heavy penalties and vexatious domiciliary visits the Apothecaries themselves, while their more quiet and fortunate neighbours, the Druggists and Chemists, may practise the art and mystery of the profession, *in all its branches*, with perfect impunity! Certes this act exhibits a magnificent specimen of judgment and acumen on the part of the committee who superintended its progress through the House.

Mr. Alcock inveighs strongly against that clause which imposes fine, and even forfeiture of practising certificate, on the apothecary who refuses to mix, compound, give, or administer the prescriptions of the physician.

"Really," says Mr. A. "the vassalage of the barbers and the barber-surgeons of antiquity, to their lords and masters the physicians, could not be more submissive than that of the humble apothecary of the present day who may happen to be *honoured* with the commands of the M.D.'s of London, Oxford, and Cambridge." 109.

We suspect that, as times go, it will but rarely happen, that the physician shall *force* the apothecary to make up his prescriptions—or that the apothecary shall consider it a crying evil when the said prescriptions are sent to them instead of the chemists. In fact, one of the complaints of the general practitioner against the physician rests on this very ground —the practice of sending prescriptions to the chemist instead of the apothecary.

In respect to the advantages derivable from the act, on the score of prohibiting improper or unqualified practitioners from exercising their art, or rather their ignorance, on the community, our author remarks—we fear with too much justice—that—

"It cannot be mentioned without regret, that the effect of these clauses has hitherto been too much resembling that of the cobweb, which entangles the small flies, and allows the greater to break through it. The very few prosecutions that have been commenced, during a period of more than seven years, have been against individuals whose extreme ignorance must have been a tolerable guarantee of their insignificance; and it may be questioned whether the entire practice of all the individuals thus prosecuted was equivalent to that of a single dispensing druggist in full trade in the metropolis." 115.

But although this act leaves the druggists and chemists to do just as they please, it must not be forgotten that it provides for a very fair professional education on the part of the surgeon-apothecary who regularly enters the profession as such. It may here be as well, for the information of a large proportion of our readers, to give a short extract containing the most recent regulation issued by the Court of Examiners at Apothecaries' Hall.

"'The Court of Examiners chosen and appointed by the Master, Wardens, and Assistants of the Society of Apothecaries, of the city of London, in pursuance of a certain Act of Parliament, "for better regulating the *Practice* of Apothecaries throughout England and Wales," passed in the 55th year of the reign of His Majesty King George the Third, have determined:

"'That every person who shall be admitted to an examination for a certificate to practise as an apothecary, shall be required to produce

"'Testimonials of having served an apprenticeship of *not less* than *five years* to an apothecary; of having attained the full age of *twenty-one years*, and being of a good moral conduct.

"'N.B. Candidates for examination are requested to take notice, that in future the production of their articles of apprenticeship, where such articles are in existence, will be considered indispensably requisite to examination; but, in any case, where such articles shall have been lost, or from any other cause may not be capable of being produced, it is expected that the candidate shall bring forward very strong testimony to prove that he has served such an apprenticeship to an apothecary, as the Act of Parliament directs.

" ' He is expected to possess a competent knowledge of the Latin language, and to produce certificates of having attended not less than

" ' Two courses of lectures on anatomy and physiology :

" ' Two courses of lectures on the theory and practice of medicine:

" ' N.B. After the 1st of January, 1823, no testimonial of attendance on lectures on the principles and practice of medicine, delivered in London or within seven miles thereof, will render a candidate eligible for examination, unless such lectures were given, and the testimonial is signed by a Fellow, Candidate, or Licentiate of the Royal College of Physicians.

" ' One course of lectures on chemistry; and

" ' One course of lectures on materia medica.

" ' A certificate of attendance for *six months at least* on the *medical practice* of some public hospital, or infirmary, or for *nine months* at a dispensary.

" ' The court have also determined, that the examination for a certificate to practise as an apothecary, shall be as follows:

" ' 1. In translating parts of the Pharmacopœia Londinensis, and physicians' prescriptions.

" ' 2. In pharmaceutical chemistry.

" ' 3. In the materia medica and in medical botany.

" ' 4. In physiology.

" ' 5. In the practice of medicine.

" ' Notice.

" ' Every person intending to qualify himself under the regulations of this Act, to practise as an apothecary, must give notice in writing, addressed to the clerk of the society, on or before the *Monday* previously to the day of examination; and must also at the same time deposit all the required testimonials at the office of the beadle, at Apothecaries' Hall.

" ' The Court will meet in the Hall *every Thursday*, where candidates are requested to attend at half past one o'clock.

" ' By order of the Court,

" ' London, Oct. 1, 1822. JOHN WATSON, Secretary.' " 117.

Mr. Alcock severely censures the clause in the above regulation which restricts the testimonials of medical attendance on lectures, (in London) to those granted by fellows, candidates, or licentiates of the College of Physicians. Certainly, as far as abstract right or justice is concerned, the acquisition of knowledge should be the criterion, and not the particular source whence it is derived. And it does look as if the Court of Examiners distrusted the efficacy of the examination by adding the clause so objected to by Mr. Alcock. The act declares that the examiners are authorised to examine all those who present themselves for that purpose—and, in another clause, the examination depends on testimonials of a *sufficient* medical education and good moral conduct. Unfortunately, says Mr. A. there is no definition of this *sufficiency* of medical education; but, will it be believed that an *apprenticeship to a druggist* has, upon *some* occasions, been thought a sterling qualification, while an apprenticeship and assistancy of nine years with a regular surgeon-apothecary, where extensive opportunities of information presented themselves, and were even put to good use, would not be listened to as the preliminary to an examination! These facts prove, says Mr. Alcock, that they who are appointed by law to superintend and carry the purposes of the act into full execution, may set its provisions at naught, and "go on and prosper"—if increase of gain be the criterion of prosperity. With the following sentiment we perfectly agree, as we have long been firmly convinced in our own minds of its truth.

"The increasing intelligence of the public calls for adequate attainments on the part of their medical attendants; and to this powerful source do I look, more than to any exclusive privileges of corporate bodies, for calling forth the energies and extending the usefulness of the profession." 120.

The subject of remuneration for the professional services of the surgeon-apothecary has been a fruitful, or rather a fruitless subject of dispute. It is, however, a subject of very great importance, inasmuch as the *mode* of remuneration, is, in our humble opinion, the greatest grievance of which the general practitioner has to complain, and, unfortunately, the most difficult of remedy.

"The mode of remunerating the general practitioner is not only degrading but unjust; it degrades that education on which his usefulness depends, sets it at naught, and values only the drugs, and not the skill that directed their use. It is degrading inasmuch as it holds out a temptation to furnish a larger quantity of medicine than may be absolutely required, and thereby exciting distrust; and it is unjust, inasmuch as the reward is not in direct ratio to the ability exercised, but inversely; the skilful practitioner who readily effects the removal of disease receiving the least; and the inefficient practitioner whose want of skill allows the disease to run a protracted course receiving the most. Further, the well-educated and efficient members of the profession are well aware that mere medicine is but a subordinate part of the successful treatment of disease; much must always depend upon the ability to detect and guard against the causes which have produced it: in acute disease improper food, &c. will more than counteract the best directed medicine." 122.

From all these considerations, Mr. Alcock asks, can it be doubted, that the welfare of the sick and the respectability of the practitioner would be promoted by the correction of so erroneous a system? We say it cannot be doubted. But we have given it all the consideration in our power, both practically and speculatively, without being able to point out any effectual remedy. Suppose it were established either by law or custom that the surgeon-apothecary should have half-a-crown or three shillings for his visit, independently of the profit on the medicines; would not the same objection still lie against the furnishing of the medicine? The *mercenary* practitioner would still have a temptation to send in too much, (for when was the *mercenary* man ever satisfied with the ratio of his profits?) and the patient would still have the suspicion that he was swallowing a little for himself, but far more for the apothecary. If, on the other hand, Mr. Alcock's plan be adopted, the patient will be dissatisfied on a different ground.

"Let the general practitioner then receive a moderate compensation for each consultation requiring the exercise of his skill and experience; and let him freely furnish, without further charge, the medicines that may be necessary to the recovery of his patient, considering them as part of the means of promoting the end for which he is consulted." 122.

Is it not evident that here the patient, who is generally out of humour when ill, would suspect that, as all which the practitioner sends after receiving his fee, is at his own cost, so he will neither send the medicines of a proper quality or quantity?—in fact, there would be constant grumbling about the medicines in this case. If you send bitter, or rough, or disagreeable medicine, then you are giving what is more fit for a horse than a man—if you send inert or agreeable ones, then you are exhibiting *sugar and water* to save expense and protract recovery. These illiberalities on the part of the patient are not imaginary. There is not a class of people on the face of the earth containing so large an alloy of illiberality and ingratitude as those who have been on the sick-list of the apothecary—when the bill is sent in.

It is impossible for either the physician, the surgeon, or the surgeon-apothecary, to place himself entirely out of the reach of suspicion among some of the less liberal-minded of his patients; but the nearest approach that can be made to this desirable state, on the part of the general practitioner, is to give the patient his advice and prescription (or surgical assistance) like the physician or surgeon, leaving him at liberty to get the medicines where he pleases. The great sacrifice here made is evident enough. The chemist and druggist will then have the whole of *pharmacy* in their own hands, and the revenue of the general practitioner will be greatly curtailed. On the whole, we do not see how the matter is to be amended in any other way than as is done now in many provincial towns, by *general consent among the practitioners themselves—namely*, that of sending only what the medical man conscientiously believes is necessary, and charging a moderate sum for attendance. In this case, no items should ever be stated, unless the patient peremptorily demands the statement, which would be a rare occurrence, as we know by five years' experience of the plan. The practitioners have only to agree among themselves upon this subject, and the public *must* adopt it. Indeed, the public would prefer it. But while a medical practitioner, of liberal education and enlightened views, is forced to make out a bill of parcels like a grocer or tallow-chandler, he cannot but feel a sort of degradation on his own part, and experience more or less of it on the part of the public at large.

On the subject of consultations Mr. Alcock makes some remarks. It is, and ever will be the case, that, in each class of the profession, now so extended, a number of unworthy members must always exist. There will be general practitioners who declare war against the physician, and endeavour to bar his access to any of their patients—and there will be physicians, who, regardless of the dignity of their profession, of the principles of moral conduct, or the rules of medical ethics, will take opportunities of making reflections, by word or by look, inimical to the reputation of the practitioner already in attendance. These systems of petty warfare are highly disgraceful to both parties, and but too well calculated to bring odium on the profession at large; for it is rather the dark than the bright side of the faculty that catches the attention of the public, and especially of the satirist. Still we are happy to say, that among all classes of the profession there is a large and preponderating balance on the side of exalted talent, useful learning, and genuine philanthropy—perhaps beyond what may be found in the other learned professions, though there is, unfortunately, less unanimity and co-operation to make these good qualities apparent to the world at large.

" 'That practitioners of medicine are influenced by very different motives than those which too often are rashly and ungenerously imputed to them, might be proved by innumerable examples. The following instance will rank high in the estimation of all men who have a due sense of the real importance of the medical practitioner, and of the serious and even awful responsibility which attaches to his office.

" 'The late Benjamin Tyre, of Gloucester, was a Surgeon not less distinguished for his scientific attainments than for his genuine philanthropy: amongst the manuscript papers examined after his decease there was found a Latin prayer, a language which he wrote, it appears, with great fluency and correctness, devoutly imploring of the Almighty that he might be enabled to render himself eminently useful to all who should come under his care; that he might never be permitted to yield to the temptations of pecuniary interest, nor convert into sordid gain the exercise of a profession, intended, under the sanction and blessing of an all-wise Creator, to promote his benevolent views in removing or lessening the various maladies incident to human nature.' " 133.

We are of opinion that associations of medical men are too rare in this country. We see, for example, in the College of Physicians, a bond of union established among its fellows, which induces them to act as harmoniously together as the various wheels of a watch or the sails of a ship, in the completion of one common object. This example should be followed by every class of the profession, especially in this metropolis, and in all great towns. By these associations the members would all become personally known to each other—which is a very potent preventive of illiberality—and laws and regulations might be framed, from time to time, that would highly conduce to their own benefit and the benefit of society in general. We hope this hint will, ere long, be acted on effectually.

To revert, in conclusion, to the main subject of this paper —the education of the general practitioner, we have but a few remarks to add to those already made. Every observer must have seen how much of the student's time is squandered away, during the period of his attendance on lectures in London, for want of some person to direct the path of his investigations. But a very few, comparatively, can be admitted into the houses of private practitioners—and even these derive little other advantage from such admissions than what results from the moral restraints put upon untimely hours and improper connexions. There is not one in fifty of the private practitioners who admit pupils, who is capable or inclined to direct their studies in the manner they ought to be directed. We think, then, that if a few such men as Mr. Alcock were to undertake the superintendance of pupils and their avocations while completing their studies in London, they would be of infinite service to the rising generation of the profession. This might be done at very little more expense to the students than they now incur in the irregular and uncomfortable manner in which they live in this metropolis, where their morals are too often corrupted, and their time misapplied for want of a home and a preceptor.

We take our leave of Mr. Alcock without making any apology for the freedom of our strictures on this paper. If we are not greatly deceived in our estimate of his character, he is not among those weak but hot-headed authors whose backs are set up if the slightest doubts are started of their infallibility on any given subject. He who comes before the public in any shape must expect opposition—if his lucubrations are worth perusing;—and he who censures others ought surely to make up his mind to be repaid in *kind*.

Art. 2. *Case of Chronic Inflammation of, and Accretion of the Pericardium to the Heart.* By James Johnson, M.D.

This is one of those diseases, the frequency of which is on the increase in the present day. The subject of the communication was Mr. Bewley, a dentist, in Duke Street, St. James's, who, for several months before his last and fatal illness, had been considered phthisical, as he laboured under cough without expectoration, febrile movements in the system, and progressive emaciation. "Occasionally, however, he was attacked with arthritico-rheumatic affections of the joints, at which times the pulmonic phenomena almost entirely disappeared." Shortly before Dr. Johnson saw him, he had had a smart attack of thoracic inflammation, and was judiciously treated by active depletion under Dr. Golding, who called in Dr. J. in consultation on the present occasion. A sudden translation had just taken place to the joints, and the pulmonary symptoms had, as on several other occasions, almost entirely disappeared. The colchicum was ordered, and in three days the patient declared himself nearly free from complaint, being able to sit up, and in high spirits. In the midst of this rapid recovery he was seized with apoplexy and paralysis of the *right* side. By bleeding, blistering, cupping, purging, and sinapisms, sensibility was restored, and in two or three days he knew his friends, and spoke, though with some difficulty. He continued to give hopes of recovery from the immediate effects of apoplexy for six days, when he fell back again, became comatose, and died about a week from the apoplectic seizure.

*Dissection.* The vessels of the head were gorged with blood, and there was some effusion between the tunica arachnoidea and pia mater. In making the incision for the centrum ovale, we came to a rent in the *right* posterior lobe of the cerebrum, containing a clot of blood, of about an ounce. The laceration communicated with the lateral ventricle of that side, which was filled with blood, as also the other lateral ventricle, the third, and the fourth ventricles.

"In the chest, the lungs might be pronounced sound. There were adhesions between the pleura costalis and pleura pulmonalis of the right side, and an effusion of a few ounces of serum in the left cavity of the chest. The pericardium and parietes of the heart were so completely accreted together by chronic inflammation, that the scalpel could not separate them without cutting one or other structure. There was no other organic lesion to be seen." 137.

Dr. Johnson states, that during the time he had attended Mr. Bewley, he always found a strong action of the heart in the proper region, *but no pulsation in epigastrio.* The pulse was regular, but generally tensive, even after considerable evacuations from the vascular system. In this case it will be observed that the hemiplegia was on the *same side* as the ruptured vessel in the brain, and clot of blood effused. It is true, the blood had also got into all the ventricles, but why should the hemiplegia have been on one side only, and that on the same side as the extravasation, which is contrary to the general rule? In fact, there is great mystery in the pathology as well as physiology of the brain.

In respect to the accretion of the heart and pericardium, it may be remembered that authors, and particularly Burns, have laid down pulsation in epigastrio as a distinctive mark. We have opened a considerable number of patients who have had this accretion, and have found the abovementioned mark as often absent as present. We will just glance at a case which we examined, while drawing up this article, in presence of Dr. Nuttall, and Mr. Freeman of Spring Gardens. A fine young gentleman of Liverpool had been affected with cough, expectoration, and palpitation of the heart for several years, and it was supposed that he was in a state of incipient phthisis from repeated thoracic inflammations, which had been very properly treated by his medical attendants in the country. In the spring of the present year, he was advised by his physician to try change of air, and accordingly he came, with two of his sisters, to Hastings. There he got so much better in the course of a few weeks that he determined to return home. Still he had much shortness of breathing on using exercise, with palpitation of the heart, and a very quick hard pulse. In the latter end of May he travelled outside of the stage from Hastings to London; but on arriving at Hatchett's Hotel, he was so ill from the motion of the coach, that the writer of this article was sent for, late at night, to see him. His pulse was 120 and peculiarly hard—breathing laborious—cough troublesome—face flushed—action of the heart perceptible, even by the eye, over a space of eight or ten inches in diameter of the thorax. The succussion of this organ could be distinctly felt by the hand over great part of the *right side* of the chest —a circumstance which convinced the reporter that there was enlargement of the heart, with either condensation of the right lung, or effusion in that side. The thorax, all over the right side, sounded like a piece of solid wood, except for a small space below the clavicle. The *left* side sounded well, except just over the region of the heart, where the sound was duller than natural. The expectoration was copious and glairy, with some specks of a purulent appearance. It was stated to his sisters that inflammation of the chest existed, which might or might not be got under; but that there was organic disease of the heart and other parts, which must inevitably prove fatal at no distant period—most likely during the present attack. It is unnecessary to detail the depletive and other measures which were used. They subdued the immediate and pressing symptoms of inflammation; but the cough, palpitation, expectoration, and laborious breathing could not be checked, and he died fifteen days after his arrival in town. Dr. Nuttall and Mr. Freeman were present when we examined the body, and we stated precisely what has been stated above—that we considered the disease to be enlargement of the heart, with condensation or effusion in the *right* side. Percussion in the dead body gave the same indications as in the living, of which the abovementioned gentlemen satisfied themselves before we put a scalpel on the corpse. On opening the thorax, marks of recent inflammatory action were conspicuous all over the pleura. The heart was nearly double its usual size, and most firmly accreted to its pericardium in every part, so that no free space was to be seen. The lung of that side was sound. The lung of the right side was completely hepatized, except a small portion under the clavicle. There was better than half a pint of bloody effusion on the right side. No other lesion of any consequence was observable.

Here a question might be asked—which was prior in its commencement—the hepatization of lung, or the enlargement of the heart, with accretion of the pericardium? We would say that the two last affections were of longest standing.

But here we must close this article, reserving for our next number a consideration of the other important papers in this volume.

